# The Human Skin Volatolome: A Systematic Review of Untargeted Mass Spectrometry Analysis

**DOI:** 10.3390/metabo12090824

**Published:** 2022-09-01

**Authors:** Anuja Mitra, Sunyoung Choi, Piers R. Boshier, Alexandra Razumovskaya-Hough, Ilaria Belluomo, Patrik Spanel, George B. Hanna

**Affiliations:** 1 Department of Surgery and Cancer, Imperial College London, Hammersmith Hospital, London W120NN, UK; 2 J. Heyrovský Institute of Physical Chemistry of the Czech Academy of Sciences, 182 23 Prague 8, Czech Republic

**Keywords:** volatile organic compounds, skin, mass spectrometry, translational science, healthy human volatolome, exogenous compounds

## Abstract

The analysis of volatile organic compounds (VOCs) can provide important clinical information (entirely non-invasively); however, the exact extent to which VOCs from human skin can be signatures of health and disease is unknown. This systematic review summarises the published literature concerning the methodology, application, and volatile profiles of skin VOC studies. An online literature search was conducted in accordance with the preferred reporting items for systematic reviews and meta-analysis, to identify human skin VOC studies using untargeted mass spectrometry (MS) methods. The principal outcome was chemically verified VOCs detected from the skin. Each VOC was cross-referenced using the CAS number against the Human Metabolome and KEGG databases to evaluate biological origins. A total of 29 studies identified 822 skin VOCs from 935 participants. Skin VOCs were commonly sampled from the hand (*n* = 9) or forearm (*n* = 7) using an absorbent patch (*n* = 15) with analysis by gas chromatography MS (*n* = 23). Twenty-two studies profiled the skin VOCs of healthy subjects, demonstrating a volatolome consisting of aldehydes (18%), carboxylic acids (12%), alkanes (12%), fatty alcohols (9%), ketones (7%), benzenes and derivatives (6%), alkenes (2%), and menthane monoterpenoids (2%). Of the VOCs identified, 13% had putative endogenous origins, 46% had tentative exogenous origins, and 40% were metabolites from mixed metabolic pathways. This review has comprehensively profiled the human skin volatolome, demonstrating the presence of a distinct VOC signature of healthy skin, which can be used as a reference for future researchers seeking to unlock the clinical potential of skin volatolomics. As significant proportions of identified VOCs have putative exogenous origins, strategies to minimise their presence through methodological refinements and identifying confounding compounds are discussed.

## 1. Introduction

The emissions of volatile organic compounds (VOCs) from the human body offer a non-invasive method for detecting and monitoring health and disease states. Although the skin is the second largest contributor to VOC excretions in humans, there is limited understanding regarding the optimal methods for their capture, association with (patho)physiological states, and potential clinical utilities [1,2].

The aggregate of VOCs emitted from the skin, otherwise known as the ‘skin volatolome’, represents a complex mixture of compounds that are generated through systemic or local metabolic processes [3,4]. Skin VOCs are derived from sweat and sebum produced from sebaceous glands [5] and are distributed via systemic circulation following endogenous site-specific production [6]. VOCs may also be generated from symbiotic bacteria that colonise the skin surface and metabolise substrates in sweat/sebum [7]. Within this mixture, the ability to differentiate between VOCs of either endogenous or exogenous origins remains a particular challenge [8,9]. Owing to the skin’s inherent position as an interface between systemic and ambient environments, it may be uniquely influenced by VOCs arising from either of these fractions or a combination of both. In general, those investigating the clinical roles of VOCs have sought to eliminate compounds presumed to be of exogenous origins by limiting sources of contamination during sample collection and handling and through the exclusion of exogenous VOCs during data processing. 

Skin VOCs are of great importance in numerous scientific, environmental, and medicinal fields. The human skin volatolome has been applied in search and rescue operations and human trafficking scenarios that rely on the detection of common VOCs, such as ketones and alcohols to identify individuals [10]. Skin volatomics have also been studied in the ecology of blood-sucking vectors of human diseases [11] and used in the design of perfumes and personal care products [12]. Personalised skin volatolomics may be of greater relevance in medicinal fields to enable the non-invasive detection of changes in an individual’s health status. A particular strength of skin volatolomics may lie in scenarios that require serial or longitudinal samplings. In the case of response to therapy and drug monitoring, the ability of the skin to excrete exogenous metabolites or surrogate markers of the systemic metabolism may be a specific advantage. An improved understanding of the profiles of VOCs emitted from the skin (and how these change in pathophysiological states and interact with drugs) will support greater confidence in clinical applications of skin volatolomics. 

The aim of this systemic review was to critically appraise skin VOCs in terms of their endogenous/exogenous origins and their association with health and disease. To achieve these aims, we performed a systematic appraisal of the published evidence concerning VOCs emitted from human skin identified using untargeted mass spectrometry methods. Untargeted methods were selected as they permit the evaluation of all detectable compounds within a sample [13]. This approach is suitable for novel discovery studies where candidate VOCs are unknown. Studies that reported on skin VOCs using targeted mass spectrometry methods where a pre-determined list of compounds was selected for analysis were excluded from this review [14,15,16,17]. There may be a role for these methods once skin VOCs of interest are identified and linked to health and disease states. Specific emphasis was given to scientific gaps in this field, such as methods used for VOC capture and analysis as well as the tentative biological origins of reported VOCs. 

## 2. Materials and Methods

### 2.1. Search Methods

An electronic literature search was performed in accordance with the recommendations of the preferred reporting items for systematic reviews and meta-analysis guidelines (PRISMA) to identify studies reporting skin VOCs using untargeted discovery methods. Searched databases included Medline (1946 to 5 June 2022), Embase (1947 to 5 June 2022) via the Ovid platform, and the Cochrane Library. The following terms were used in the search strategy: skin, VOCs, biomarkers, metabolomics, adult, metabolic profiling, and mass spectrometry. All variations in the spelling, including truncated search terms using wild card characters and the “related articles” function, were used in combination with the Boolean operators AND OR. Full details of the search strategy can be found in Supplementary File S1. Reference lists of qualified articles were screened and two independent reviewers, (A.M. and S.C.) reviewed the full texts for eligibility. Only full-text original articles written in the English language were considered. Review articles, conference abstracts, and animal and cell studies were excluded. A third reviewer (I.B.) was consulted when disagreements in the study inclusion arose. 

### 2.2. Quality Assessment

Methodological and reporting standards of the included studies were evaluated using the Quality Assessment of Diagnostic Accuracy Studies-2 (QUADAS-2) and the Standards for Reporting of Diagnostic Accuracy Studies (STARD). The QUADAS-2 score rates the quality of each paper based on four domains: patient selection, index test, reference standard, and the flow of patients through the study. The STARD tool assesses the quality of reporting of the included studies using a 30-item checklist that links each item to potential sources of bias, variability, and limitations of the applicability of results to other settings. Both quality assessment tools were independently conducted by two reviewers (A.M. and S.C.) to rate all included studies. 

### 2.3. Outcome Measures

The primary outcome measure was chemically verified VOCs detected from human skin. Data extracted from studies were publication year, country, study design, participant number, disease status, MS platform, sampling technique, sampling material, time and storage, anatomical location for sampling, number of VOCs detected, and quality control method(s). The reported skin VOCs were corroborated in accordance with the Kyoto Encyclopedia of Genes and Genomes (KEGG) pathway and the Human Metabolome Database (HMDB) using their CAS numbers. 

## 3. Results

**Figure 1:**
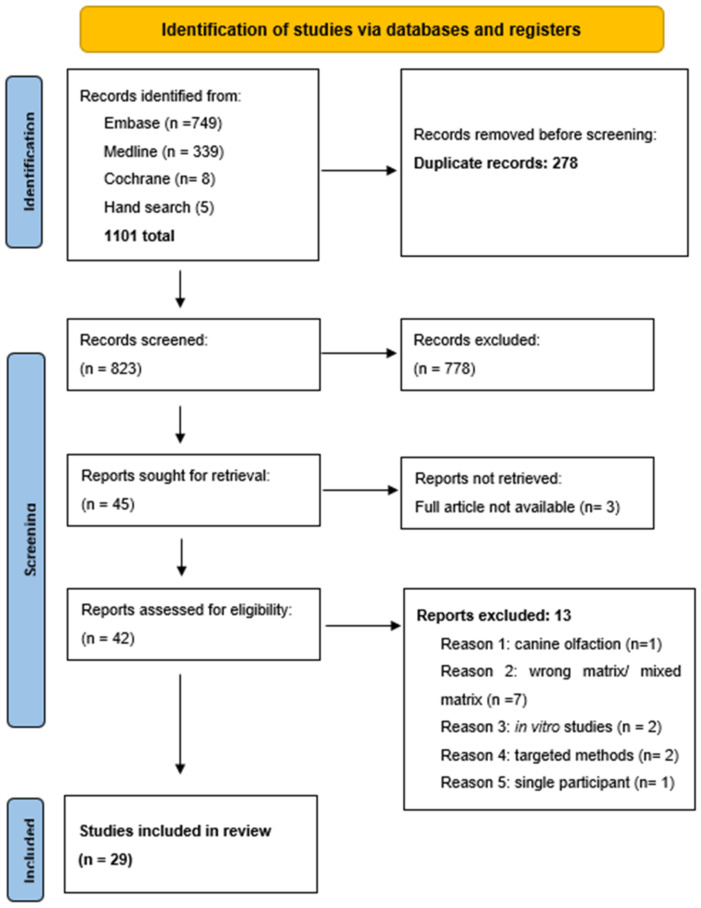
PRISMA flow chart of literature.

The systematic literature search identified 999 original studies. Following screening and assessment for eligibility, a total of 29 full-text articles reporting results from 935 participants met the inclusion criteria. Full details of the included studies are presented in Table 1 and the PRISMA flowchart is provided in Figure 1. Twenty-two studies profiled skin VOCs from disease-free (healthy) participants and seven studies investigated skin VOCs associated with a range of disease states (wound infections, *n* = 4; melanoma, *n* = 2; mixed visceral adenocarcinoma, *n* = 1).

### 3.1. Quality Assessment Outcomes

Of the included studies, there was high applicability to the review question and an overall moderate risk of bias. Outcomes of STARD and QUADAS-2 assessments are summarised in Supplementary File S2. Major sources of bias were related to most studies being non-randomised cohort studies without appropriate control groups and with significant heterogeneities in the study designs. STARD scores ranged from 12 to 27 (/30) with a mean of 20 ± 3.4, indicating that reporting standards were often inadequate.

### 3.2. Method of Skin VOC Capture

Skin VOCs were sampled from a broad range of body sites, including the hand (n = 9), forearm (*n* = 7), back (*n* = 3), wrist (*n* = 2), ankle (*n* = 2), forehead, (*n* = 2), foot (*n* = 2), axilla (*n* = 2), whole body (*n* = 2), navel (*n* = 1), leg (*n* = 1), and chest (*n* = 1). 

Methods used to capture skin VOCs could be broadly classified as either: (i) the direct analysis of gas above or adjacent to a skin region or (ii) indirect capture of VOCs on to an absorbent material placed directly on or close to the skin surface. 

Three studies directly analysed VOCs within an enclosed chamber that contained either the whole subject or a region of the body. The analytical platforms used by these studies were capable of direct sample injections, such as the selected ion flow tube mass spectrometer (SIFT-MS). 

Twenty-six studies captured skin-associated VOCs with a range of absorbent materials, including: solid phase microextraction (SPME) (*n* = 10), polydimethylsiloxane (PDMS) (*n* = 7), cotton [6], Tenax/Carboxen [1], and glass beads (*n* = 2). Fifteen studies used contact-based methods where absorbent materials were placed directly on the skin allowing for passive transfer of VOCs. The remaining studies sampled gas that was directly adjacent to a region of skin held within a contained environment. In studies where non-contact-based methods for VOC capture were applied, VOC transfer was achieved via either passive absorption or active aspiration of gas onto the sorbent material. The duration of passive transfer and the volume of active aspirate varied significantly between studies. 

Following capture, VOCs contained within sample materials were released using thermal desorption prior to volatile analysis using a range of analytical platforms (Table 1).

### 3.3. VOC Analysis

Various analytical platforms were used for untargeted VOC analysis. One-dimensional gas chromatography–mass spectrometry (GC–MS) was most commonly used (*n* = 23) followed by two-dimensional GCxGC–time of flight–MS (GCxGC-ToF-MS, *n* = 3) and GC–ion mobility spectrometry (GC-IMS, *n* = 1). These methods utilised thermal desorption to release skin VOCs from the sorptive material into the GC. Three studies used the direct injection of the volatile headspace into the GC sampler for analysis. One study used a selected ion flow tube-MS (SIFT-MS) and a selected reagent ion-MS (SRI-MS), sampling the volatile headspace from an enclosed body chamber in real-time. 

### 3.4. The Human Skin Volatolome

A total of 822 unique VOCs associated with human skin were identified from the included studies (Supplementary File S3). Although the chemical classes across studies were consistent, the numbers of reported VOCs between studies varied significantly. Several factors influenced VOCs that were detected, such as the use of adsorptive versus direct methods of VOC capture and analysis, which generated an average of 22 and 172 VOCs, respectively, and the skin patch material (where PDMS patches generated an average of 35 compounds) versus cotton patches (which produced 77 compounds). 

**Figure 2:**
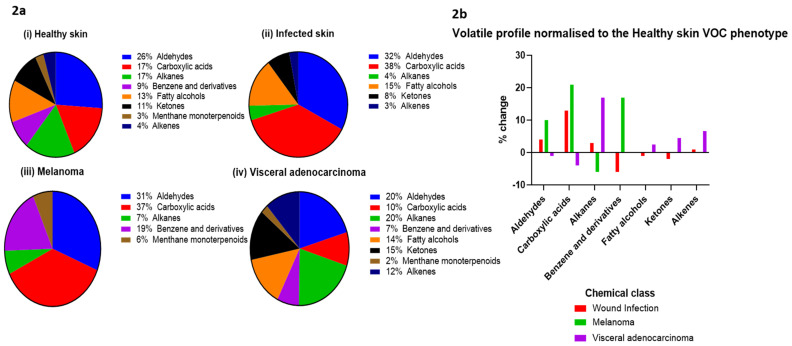
The composition of the human skin volatolome in health and disease. (**a**) The composition of the skin volatolome from the top eight chemical classes from (i) healthy skin, (ii) infected skin, (iii) participants with melanoma, and (iv) patients with mixed visceral adenocarcinoma. (**b**) Percentage change in the skin VOC profile normalised to the healthy skin profile in a range of disease states.

A total of 22 studies evaluated VOCs from regions of disease-free skin, identifying 689 compounds from 32 different chemical classes, the most common being: aldehydes (18%), carboxylic acids (12%), alkanes (12%), fatty alcohols (9%), ketones (7%), and benzenes and derivatives (6%) (Figure 2a). The most common VOCs detected from healthy skin were decanal (*n* = 21 studies), nonanal (*n* = 20), octanal (*n* = 18), heptanal (*n* = 15), benzaldehyde (*n* = 15), 6-methyl, 5-hepten-2-one (*n* = 11), and acetone (*n* = 9). 

**Figure 3:**
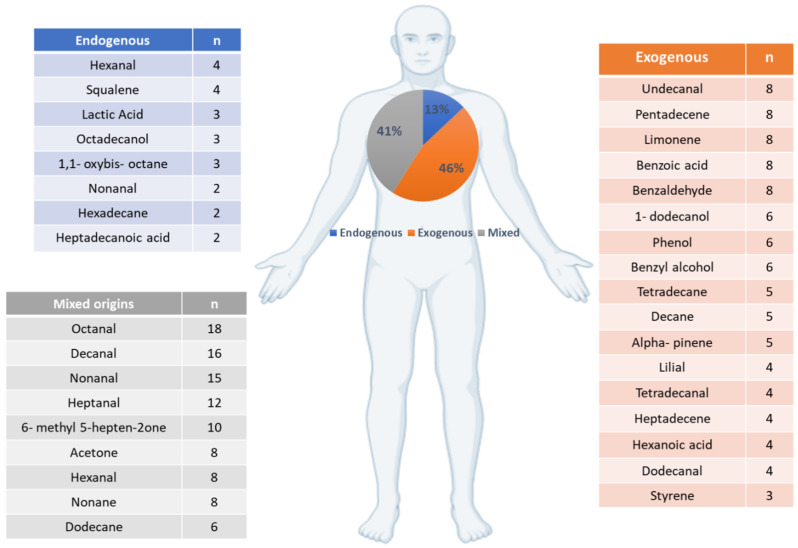
The putative biological origins of skin volatile organic compounds emitted from human skin. The putative biological origins of the compounds representing the healthy human skin volatolome, grouped into endogenous, exogenous, and mixed origins. The most common compounds in each category are included; n represents the number of papers each compound was identified in.

The putative biological origins of skin VOCs of healthy skin were evaluated by matching individual compounds using their CAS numbers against the KEGG and Human Metabolome databases. Results demonstrated 13% of compounds likely had endogenous origins, 46% had exogenous origins, and 41% were present in mixed pathways. The most commonly detected compounds from each group are summarised in Figure 3.

Seven studies investigated the skin VOC profiles of cutaneous disease states (wound infection *n* = 4, melanoma *n* = 2, and systemic adenocarcinoma *n* = 1) (Figure 2b). Early results demonstrate a difference in the skin VOC profiles in disease.

From the four papers investigating the VOCs profiles of infected skin, a total of 86 VOCs were reported, the most common being nonanal, tetradecanal, acetic acid, 2-ethyl 1-hexanol, 6, 10-dimethyl-2-undecanone, and 1, 1-oxybis octane. The proportions of carboxylic acids and aldehydes were significantly higher with infections compared to healthy participants (25% vs. 12% and 22% vs. 18%, respectively) and there was a reduction in the proportion of alkanes emitted from the skin in infected wounds (3% vs. 12%) (Figure 2).

A single study investigated skin VOCs from 32 patients with mixed visceral adenocarcinoma (lung, prostate, gastric, kidney, head and neck, pancreas, and colorectal). Of the 52 VOCs that were reported, compounds from low molecular weight non-polar chemical classes, such as alkenes, alkanes, and ketones were high in cancer patients in comparison to healthy patients. In comparison, carboxylic acids were lower in the cancer group, reflecting a shift in the VOC skin signature in disease states that were detected non-invasively.

## 4. Discussion

This is the first review to comprehensively summarise the evidence relating the human skin volatolome and its relation to health and disease. The principal findings were: (i) the identification of 822 VOCs from the skin volatolome; (ii) recognition of those compounds with potential endogenous origins; (iii) the association of specific VOCs with cutaneous and systemic disease states, and (iii) significant heterogeneity in the study design and methodological quality. 

The study of VOCs and their role in disease detection and monitoring has rapidly evolved over the last two decades, supported by technical advancements in analytical methods, emphasis on compound identification, elucidation of biological origins, and an increasing appreciation of the importance of standardisation and quality controls. Whilst the majority of clinical volatolomics research has focused on VOC detection within exhaled breath, other contributors to the volatolome are recognised, including skin and urine [1,43,44]. In all cases, detection of VOCs occurs entirely non-invasively. VOC detection from the skin is particularly attractive owing to the potential to detect both locally occurring as well as broader systemic changes in the metabolism, the ease of accessibility, and the high patient acceptability of this nature of sampling. 

This review identifies a core skin VOC signature of human health. This signature is predominantly composed of VOCs from the chemical classes of aldehydes, carboxylic acids, ketones, and fatty alcohols. Generally, acetone, acetaldehyde, 6-methyl-5-hepten-2-one, nonanal, and decanal were ‘stable’ volatiles consistently detected at high emission rates. Aldehydes are considered to be terminal products of local and systemic lipid peroxidation reactions of cellular membranes and lipid droplets generated from oxidative stress reactions [45,46]. It is known that levels of oxidative stress are enhanced in tumour microenvironments; therefore, this alludes to the possibility of volatile aldehydes being cancer biomarkers. A higher concentration of aldehydes is demonstrated in the exhaled breath of patients with oesophagogastric adenocarcinoma [47] and early results in skin VOCs have revealed higher levels of aldehydes from patients with mixed visceral adenocarcinomas in comparison to healthy participants strengthening the link between VOCs and human pathophysiology [48,49]. VOCs belonging to the ketone chemical class, such as acetone, 2-butanone, 3-buten-2-one, and 6-methyl-5-hepten-2-one were consistently detected across the healthy skin volatolome. Owing to the reliability of their emissions from human skin, these compounds have been used in studies investigating the human scent of trapped individuals in search and rescue operations [24].

Carboxylic acids were the second most common chemical class of skin compounds. Their presence may be explained by the ubiquitous sebum layer, which is rich in waxy lipids, such as ceramides that coat and protect healthy skin whilst serving as anti-microbial defence barriers [50]. Oxidation of these lipids is the main mechanism to produce smaller chain unsaturated carboxylic acids, such as 9-decenoic and 10-undecenoic acids detected in the skin VOC profile. Conversely, damage to the sebum layer through injury or barrier disruption has been shown to reduce the proportion of carboxylic acids released [23]. Changes in skin carboxylic acids have also been observed in initial studies using skin VOCs to diagnose pathophysiological states, such as infection [19,20,23,37] and melanoma [42]. Researchers demonstrated an increase in levels of carboxylic acids, particularly in two compounds, lauric acid and palmitic acid in melanoma. These compounds are recognised endogenous metabolites generated through lipid biosynthesis within the tumour microenvironment and are also present in numerous personal care products [51]. Conversely, VOCs, such as dimethyl disulphide and dimethyl trisulfide, are not endogenously produced by normal melanocytes, highlighting a potential use as diagnostic biomarkers for melanoma, which reflects the increased methylation process in melanoma. 

The proportion of carboxylic acids was raised in the volatolome of infected skin. Bacteria are known to be a rich source of VOCs and prokaryotic lipids are considered to be more metabolically complex containing longer and uneven branched-chain fatty acids tails that can be sources of the production of carboxylic acids [19]. Furthermore, bacteria convert long-chain non-volatile compounds into short-chain volatile compounds, which may account for the increase in the proportion of fatty acids emitted in infection. 

At the present time, there is limited understanding of the optimal methodology for capturing and analysing skin VOCs. This is emphasised by the current finding that no two studies share a directly comparable methodology. Except for a small number of studies that have employed direct injection analytical methods, most studies could be broadly categorised as those that used absorptive agents placed either in direct contact with the skin or near the skin. There was a preponderance towards the use of contact-based absorption over non-contact methods, despite the latter appearing to generate more VOCs. This may be explained by the fact that, with non-contact-based sampling, the participant is required to be present for the entire sampling time. Conversely, the application of an absorptive skin patch is easy, self-administrable, and can be performed quickly in the community permitting a ’hub and spoke’ method of sampling [52]. Skin patches are commonly composed of PDMS or cotton. Whilst PDMS may be suited to VOC capture due to its affinity for low molecular weight volatiles, cotton patches are cost-effective, scalable, and appear to capture more VOCs than PDMS [25]; however, this may be due to higher levels of contamination at the baseline of cotton patches, which are reflected in the number of VOCs released. 

GC–MS was the most common analytical platform used for skin VOC analysis owing to its ability to detect low molecular weight compounds of polar and non-polar nature with high sensitivity and specificity. Several recent publications have demonstrated the benefit of using multidimensional gas chromatography (GCxGC-ToF-MS), which is reported to increase chromatographic resolution, leading to a reduced number of coeluting peaks [11,21,26]. When coupled to mass spectrometry, this produces cleaner mass spectra, increasing confidence in compound identification when using commercial mass spectral libraries, such as NIST and WILEY. These platforms are well-suited to untargeted biomarker discovery studies, and when used in combination with an absorptive agent can be adapted for high throughput offline analyses. In the future, accurate and affordable instruments capable of real-time analyses of targeted skin VOCs using analytical platforms, such as the SIFT-MS, as well as wearable sensors, may provide additional opportunities to widen the access to volatile biomarker assessments in clinical practice. 

Exogenous VOCs are present in all biological sampling matrices; however, this is particularly troublesome for skin VOCs due to their continual contact with the environmental exposome [53]. This was corroborated in the current review, which identified that a large proportion of VOCs detected from skin were deemed exogenous or from mixed metabolic pathways. Compounds such as limonene, undecanal, and 1-dodecanol are commonly detected exogenous skin compounds that are regularly used in food flavouring, preservatives, and fragrances [54,55]. Compounds such as phenol are abundantly expressed in the environmental exposome with high levels in urban areas due to heavy motor traffic emissions and are metabolites of common medications, such as aspirin [56,57]. Significant proportions of skin compounds also have mixed biological origins, such as benzoic acid, which is a widely used fungistatic compound (used as a food preservative); however, it is also produced endogenously as a by-product of phenylalanine metabolism in gut bacteria [58]. It is also important to identify VOCs that are endogenously produced, as well as being terminal metabolites of drugs (such as xenobiotics) and other common medications [59]. Although it is impossible to eliminate all exogenous VOCs, efforts can be made to minimise their presence. Such measures include asking participants to refrain from the use of personal care products prior to sampling, sampling in well-ventilated environments, and careful selection of the anatomical location of skin sampling [60]. One previous study identified that dimethyl sulphone, a constituent of many perfumes, occurred in higher concentrations on the forearms of healthy subjects compared to the backs. In comparison, the backs of healthy subjects had higher levels of hexyl salicylate and hexyl cinnamaldehyde that are found in shampoo, which reflects how participants use consumer products [6]. 

In view of the significant heterogeneity in the observed methodologies for skin VOC sampling and the potential for this to impact future findings, a summary of potentially important methodological considerations that can be used to standardise skin-based volatile sampling is provided in Table 2. 

An important finding (and limitation) of the review is that most of the research concerning the skin volatolome is related to healthy subjects. There is currently insufficient evidence to draw any meaningful conclusions relating to skin VOCs and specific disease states using untargeted methods. Those studies that were identified did provide tentative evidence of a link between skin VOCs and both cutaneous and systemic disease; however, further quality controlled, and methodologically standardised studies are required to validate these findings. It is also important to acknowledge that this review included studies using untargeted methods in order to report the broader skin volatolome. Studies that investigated skin VOCs in health and disease, such as the one by Trivedi et al., which investigated skin VOCs in Parkinson’s disease with targeted methods, and those using direct online platforms, such as SESI-MS and SIFT-MS, were excluded [13]. Targeted methods can arguably provide greater sensitivity for VOC detection and can have a complementary role alongside untargeted techniques once a list of candidate VOCs is identified. 

Efforts should also be made to better understand the mechanism(s) and kinetics whereby VOCs are released by the skin and the relative contributions of both local and systemic metabolism. Other important contributing factors, such as the impact of local physiology and the role of the skin microbiome on VOC production, should be investigated in greater detail. Although several studies researched the influences of different anatomical sites and skin infection on the VOC profile, no individual study investigated the influence of skin-specific bacterial species on VOC production. Considering resident microbiota are known to be plentiful sources of VOCs, more work is needed to understand their role in producing unique skin volatile profiles. 

## 5. Conclusions

In conclusion, this review provided a comprehensive overview of the current literature concerning the detection of VOCs from human skin. A major contribution of this work is the description of the healthy skin volatolome and its classification based on the putative endogenous and exogenous origins of the compounds. To develop future studies and translate results to clinical practice, the challenges associated with skin VOC analysis that are summarised in this review should be addressed. 

## Figures and Tables

**Table 1:** Study characteristics, methods of skin volatile organic compound (VOC) sampling, and analytical platform used.

	Sampling Location	Patient Cohort	Number of Participants	Analytical Instrument	Method of Skin VOC Collection	Sorbent Material	Sampling Time (Hours)	Skin Cleaning Prior to Testing	Sample Storage Temperature		Sample Storage Time	Number of Significant Skin Compounds	Reference
**Monedeiro 2020**	Lower back	Visceral adenocarcinoma	64	GC–MS	SPME direct contact	Cotton pad	48	Sampling location cleaned with isopropanol	−20		Immediate processing	72	[18]
**Ashrafi 2020**	Upper arm	Healthy	5	GG–MS	Direct wound headspace onto TD tubes and PDMS direct contact patches	Custom 3D polylactic funnels to create headspace over the wound for headspace, PDMS patches	0.5	No use of exogenous products for 24 h before sampling	n/a		Within 24 h	10	[19]
**Vishinkin 2021**	Inner arm and chest	Active TB infection	461	GC–MS	Static headspace no concentration step	PDMS patch, porous polymeric pouches containing poly (2,6-diphenyl phenylene oxide)	1	No smoking for 30 min before	4		Up to 8 months	6	[20]
**Roodt 2018**	Ankle, wrist	Healthy	2	2D GC ToF MS	PDMS bracelet directly into GC	PDMS bracelets	4	No effort to control environmental factors	−80		Max 72 h	87	[21]
**Wooding 2020**	Ankle, wrist	Healthy	20	GCxGC-ToF-MS	PDMS sampler directly analysed in GC	PDMS patch	1	Medical-grade alcohol cleansing pads (70% isopropanol) used to clean the skin surface	4		Within 48 h	39	[11]
**Grabowska-Polanoska 2017**	Forearm	Healthy	8	GC–MS	(a) Forearm headspace using Cellulose bag (60x 30 cm)	(b) Cellulose sachets filled with active carbon (patch)	Carbotrap X	2	Y (refrain from perfume, deodorants, fragrance-free soaps)	n/a	Immediate processing	49	[22]
**Duffy 2017**	Forearm	Mechanical barrier disruption	7	GC–MS	Static headspace SPME		50/30 ym DVB/Carboxen/PDMS SPME, held in glass vial	0.25	Y (no cosmetics on the area on the day, wash with olive-based soap)	n/a	Immediate processing	37	[23]
**Mochalski 2014**	Hand, forearm	Healthy	31	GC–MS	SPME static headspace		Nalophan bags, 75 ym Carboxen/PDMS	0.5	Wash arm with tap water and dry with a paper towel	immediate processing	Immediate processing	22	[24]
**Prada 2011**	Hand	Healthy	6	GC–MS	Headspace SPME (DVB/CARB on PDMS) and direct sampling with contact SPME		Cotton, rayon, polyester, wool	12	no	n/a	Immediate processing	58 (direct sampling) and 20 (headspace)	[25]
**Dolezal 2017**	Hand	Healthy	9	2D GC ToF MS	Solvent extraction from glass beads		40 glass beads (4.86 mm), hexane for solvent	0.2	Y (wash hands with non-perfume soap)	Below 7	Unknown	137	[26]
**Bernier 2000**	Hand	Healthy	4	GC–MS	TD of glass beads into GC port		2.9 mm glass beads	0.25	No specific instructions prior to collection but hand washing with the same soap on arrival	n/a	Immediate processing	233	[27]
**Kusano 2013**	Hand	Healthy	31	GC–MS	Headspace SPME		Gauze pads in a glass vial, 50/30 ym DVB/Carboxen on PDMS	0.2	Hand washing pre-collection with olive-based soap	n/a	Immediate processing	217	[28]
**Broza 2014**	Hand	Healthy	30	GC–MS	Direct dynamic headspace to TD tube		Direct headspace of VOCs onto to Tenax/Carboxen TD tube using a pump	Immediate	Washed with water for 1 min	Up to 1 week	Up to 1 week	12	[29]
**Curran 2007**	Hand	Healthy	60	GC–MS	Headspace SPME		Headspace SPME of pre-treated gauze, SPME (CAR/DVB/PDMS 50/30 ym)	0.17	Hand washing 30 s olive-based soap, washing with water, air drying	21	Immediate processing	62	[30]
**Zhuo-Min 2005**	Hand/forearm	Healthy	15	GC–MS	Static Headspace SPME		Hand-placed in stainless steel jar/glass jar, SPME 65 ym DVB/PDMS	0.5	no	n/a	Immediate processing	34	[31]
**Turner 2008**	Hand/forearm	Healthy	5	SIFT MS	Dynamic headspace		Nalophan bags	0.12	No	n/a	Immediate processing	6	[32]
**Gallagher 2008**	Forearm, back	Healthy	25	GC–MS	SPME static headspace using 50/30 ym DVB/Carboxen on PDMS		Direct headspace using a glass funnel	0.5	No	−12 (only for solvent)	Immediate processing	56	[6]
					Solvent extraction		Double distilled ethanol			n/a		49	
**Martin 2016**	Forehead	Healthy	15	GC–MS	PDMS patch		PDMS	0.5	No	−80	Stored until processing	30	[33]
**Verhulst 2016**	Axilla, hands, feet	Healthy	8	GC–MS	Static headspace of cotton pads onto TD unit		Cotton pads	0.13	No	−80	Max 21 days	4	[34]
**Dormont 2013**	Feet	Healthy	26	GC–MS	Solvent extraction		Diethyl ether/dichloromethane	0.03	Washed sampling area with water	n/a	Immediate processing	32	[35]
					SPME static headspace		50/30 ym DVB/Carboxen on PDMS on StableFlex fibres	0.75		n/a		23	
					SPME patch (direct contact)		50/30 ym PDMS-DVB/CAR	0.05		n/a		24	
					Dynamic headspace		Nalophan bags, Carbotrap, Tenax	0.33		n/a		38	
**Ruzsanyi 2012**	Navel	Healthy	7	GC–IMS	Real-time VOC collection by IMS		102 mm stainless steel pan	0.08	Avoid alcohol 12 h, no cosmetics on day	n/a	Immediate processing	10	[36]
**Thomas 2010**	Lower limb	Chronic arterial leg wounds	5	GC–MS	PDMS skin patches		Direct patch, inserted into TD tubes	0.5	Washed with distilled water 24 H before sampling	n/a	Unknown	46	[37]
**Haze 2001**	Upper back	Healthy	22	GC–MS	Dynamic headspace of T shirt		Tedlar bags, Tenax	72	Y odourless soaps/cosmetics	n/a	Immediate processing	22	[38]
**Curran 2005**	Axilla	Healthy	4	GC–MS	Static Headspace SPME		Sterile gauze, SPME DVB/Carboxen on PDMS	24	Olive oil soap for 48 h during shower	21	Immediate processing	46	[8]
**Meijerink 2000**	Forehead sweat	Healthy	14	GC–MS	Dynamic headspace		Tenax pooled sweat	3	Avoid soap/cosmetics for 24 h	n/a	Immediate processing	76	[39]
**Mochalski 2014**	Whole body	Healthy	10	SRI-ToF-MS	Body plethysmography chamber		Headspace directly loaded onto MS	1	Asked to fast for 12 h prior and refrain from using cosmetics	Immediate processing	Immediate processing	64	[24]
**Mochalski 2018**	Whole body	Healthy	11	GC–IMS	Headspace		Whole body plethysmography chamber (bodyscope)	1	Y (refrain from cosmetics for 12 h)	n/a	Immediate processing	17	[40]
**Abaffy 2013**	Melanoma biopsy samples	Melanoma	10	GC–MS	PDMS/DVB SPME headspace of biopsy		Biopsy put into headspace vials with SPME as a concentration step	1	No	n/a	Immediate processing	6	[41]
**Abaffy 2010**	Melanoma biopsy samples in headspace vials	Melanoma vs. benign nevi	20	GC–MS	SPME		PDMS-DVB	1	No	n/a	Immediate processing	9	[42]

**Table 2:** Considerations for the analysis of skin volatile organic compounds.

Workflow	Analytical Step	Considerations
Experimental design	Define intended population	Patient selection
		Separate into training and independent validation cohorts
		Define the anatomical location of skin sampling
Sample preparation	Pre-sampling	Avoid personal care products at the location of sampling for a minimum of 24 h
		Consider patient medication and use of xenobiotics
	Skin VOC collection and storage	Direct contact vs. non-contact methods
		Choice of sampling material (cotton/PDMS/SPME)
		Sampling time
		Sources of contamination
		Sample storage (direct analysis or storage at -80 freezer)
	Sampling technique	Consider the use of pre-concentration headspace step or direct injection
		Use of internal standards in patch
		QCs (spiking with internal standards to monitor and correct analytical variability)
MS analysis	Analytical platform	Online or offline platforms
		Optimisation of MS parameters
	MS data collection	Run order (randomisation) and batch effects
Data Analysis	Data pre-processing	Data deconvolution, scaling, peak alignment
		Removal of irreproducible, contaminant compounds
	Statistical analysis	Descriptive statistics
		Univariate analysis
		Multivariate analysis (e.g., PCA, PLS-DA, OPLS-DA)
		Prediction model (e.g., ROC analysis)
	Biological interpretation	Metabolic pathways (KEGG, HMDB)

## Data Availability

The data presented in this study are available within the article and the Supplementary material.

## Supplementary Materials

The following supporting information can be downloaded at: https://www.mdpi.com/article/10.3390/metabo12090824/s1.
