# Supplementary material for: The Human Skin Volatolome: A Systematic Review of Untargeted Mass Spectrometry Analysis
Source: Metabolites. 2022 Sep 1;12(9):824. doi: 10.3390/metabo12090824 (PMC9504915; doi:10.3390/metabo12090824)
Supplement: Supplementary file 1 [file metabolites-12-00824-s001.zip › metabolites-1860179-supplementary.pdf]

Supplementary File S1

Developing the search strategy

The search strategy was required to capture volatile organic compounds emanating from the human skin of adult participants using untargeted mass spectrometry methods. Review articles, conference abstracts, and animal and cell studies were excluded. The search strategy took account of the variability in the way authors describe their research and indexers index research with Medical Subject Headings (MeSH). All variations in spelling including truncated search terms using wild card characters and the "related articles" function were used in combination with the Boolean operators AND OR. Abbreviations and search terms are explained in Box 1

Box 1

Exp	Explodes a MeSH to capture more specific MeSH.
/	Indicates that the search term is a Medical Subject Heading (MeSH).
AND	Achieves a Boolean AND Combination.
OR	Achieves a Boolean OR Combination.
.mp.	Searches for the term expressed in the title, original title, abstract, and subject heading.
*	Truncation operator, searches for words beginning with the stem, e.g. epidemiol* retrieves epidemiology, epidemiological and epidemiologic.

The following databases were searched:

- Ovid MEDLINE (R) 1946 to 2022 June 05;
- Ovid Embase 1974 to 2022 June 05;
- Cochrane library from inception to 2022 June 05

Records identified from each electronic and regional database were downloaded into a separate Endnote bibliographic database, which were then combined to generate a single large Endnote bibliography containing all the records identified from all the databases in which duplicate records were removed.

Final search strategy

The final search strategy and results for the MEDLINE search is shown below:

Medline search

Embase Classic+Embase <1947 to 2022 June 05>

Number	Search Strategy	Number of articles

1	exp Human Experimentation/ or exp Humans/ or human experiment.mp. or exp Adult/	25626797
2	skin.mp. or exp Skin/	1450023
3	volatile organic compound.mp. or exp Volatile Organic Compounds/	22737
4	exp Gas Chromatography-Mass Spectrometry/ or exp Volatile Organic Compounds/ or volatile compound.mp. or exp Chromatography, Gas/	183808
5	3 or 4	184367
6	2 and 5	3903
7	exp Adult/ or adult.mp.	10819640
8	1 and 6 and 7	749

Supplementary File S2

Table S2a. Quality assessment with Standards for Reporting of Diagnostic Accuracy studies (STARD) and Quality of Diagnostic Accuracy Studies-2 (QUADAS-2). Overall, there was a moderate to low risk of bias and low concern regarding applicability of the 29 studies reviewed.

	QUADAS- 2								
		Risk of Bias				Applicability Concerns			
Study	**Overall Diagnostic Quality**	**Patient Selection**	**Index Test**	**Reference Standard**	**Flow and Timing**	**Patient Selection**	**Index Test**	**Reference Standard**	**STARD Score**
Vishinkin 2021	Good	Low	Low	Low	Low	Low	Low	Low	21
Wooding 2020	Good	Low	Low	Low	Low	Low	Low	Low	15
Ashrafi 2020	Good	Low	Low	Low	Low	Low	Low	Low	20
Monedeiro 2020	Good	Low	Low	Low	Low	Low	Low	Low	26
Roodt 2018	Fair	Low	Low	Low	Low	Low	Low	Low	19
Mochalski 2018	Good	Low	Low	Low	Low	Low	Low	Low	25
Dolezal 2017	Fair	Low	Low	High	High	Low	Low	Low	23
Duffy 2017	Good	Low	Low	Low	Low	Low	Low	Low	23
Grabowska-Polanoska 2017	Good	Low	Low	Low	Unclear	Low	Low	Unclear	18
Martin 2016	Good	Low	Low	Low	Low	Low	Low	Low	19
Velhurst 2016	Good	Low	Low	Low	Low	Low	Low	Low	23
Mochalski 2014	Good	Low	Low	Low	Low	Low	Low	Low	17
Mochalski 2014	Good	Low	Low	Low	Low	Low	Low	Low	21
Broza 2014	Good	Low	Low	Low	Low	Low	Low	Low	18
Abaffy 2013	Good	Low	Low	Low	Low	Low	Low	Low	20
Kusano 2013	Good	Low	Low	Low	Low	Low	Low	Low	19
Dormont 2013	Good	Low	Low	Low	Low	Low	Low	Low	24
Ruzsanyi 2012	Good	Low	Low	Low	Low	Low	Low	Low	27
Prada 2011	Fair	Low	High	High	High	Low	Low	Low	24
Abaffy 2010	Good	Low	Low	Low	Low	Low	Low	Unclear	12
Thomas 2010	Good	Low	Low	Low	Low	Low	Low	Low	18
Turner 2008	Fair	Unclear	Low	Low	High	Low	Low	Low	18
Gallagher 2008	Good	Unclear	Low	Low	High	Low	Low	Low	21
Curran 2007	Fair	Unclear	Low	Low	Low	Unclear	Low	Low	18
Zhang 2005	Fair	Unclear	High	High	High	Low	Low	Low	17
Curran 2005	Fair	Unclear	Low	Low	Low	Unclear	Low	Low	21
Haze 2001	Good	Unclear	High	Low	High	Unclear	Low	Low	21
Meijerink 2000	Good	Unclear	Low	Low	Low	Unclear	Low	Low	16
Bernier 2000	Fair	Unclear	High	High	High	Unclear	Low	Low	17

Page Break

Table S2b: Risk of bias and applicability concerns using QUADAS- 2

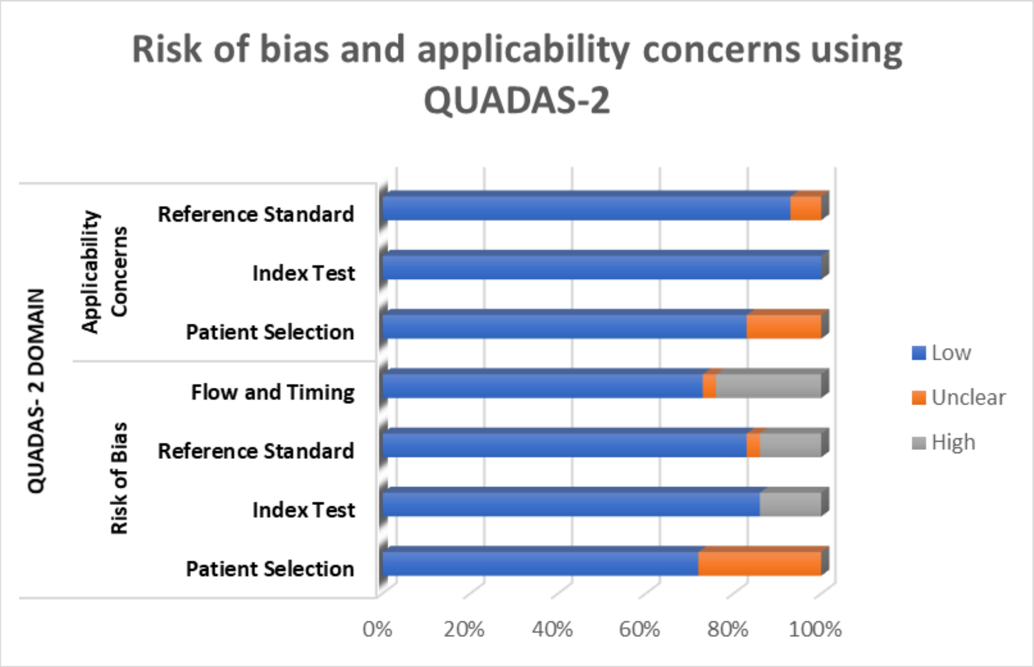

Supplementary File S3: Details of Volatile Organic Compounds Emitted from Disease Free Human Skin

Author	Year	Healthy/ Disease	Number of Identified Compounds	Compounds
Vishinkin	2021	Infection	6	Acetic acid, octanoic acid, 1-hexanol, 2-ethyl-, hexyl butyrate, toluene, cyclopropane, pentyl-
Wooding	2020	Healthy	38	Dodecanoic acid, Tetradecanoic acid/Myristic Acid, Pentadecanoic acid, Heptadecanoic acid 9,12-Octadecadienoic acid (Z,Z)-, Octanal, 2-Undecanone, 1-Octanol/Octanol/Isooctanol, 1-Nonanol, Octadecanol, 1-eicosanol, ethanol, 2-(2-ethoxyethoxy)-, ethanol, 2-(dodecyloxy)-, 1, octanol, 2-butyl, 1-heptanol, 6-methyl-, hexadecen-1-ol, trans-9-, Butyrolactone, 2,6,10,15,19,23-hexamethyl-2,6,10,14,18,22-tetracosahexaene (squalene), cyclooctatetraene, naphthalene,5-ethyl-1,2,3,4—tetrahydro, cyclobutylamine, benzonitrile, 3,5-dimethyl-, pentadecane,2-methyl-<br>hexadecane, 3-methyl, Hexadecane, 2,6,10,14-tetramethyl-, Octane, 1,1′-oxybis-, 4-cyanocyclohexane, Methyl salicylate, acetic acid, octyl ester, octadecanoic acid, 2,3, dihydroxypropyl ester, benzene butanoic acid gamma-oxo-, ethyl ester, Isopropyl Palmitate 1,2-Benzenedicarboxylic acid, bis(2-methylpropyl) ester, octyl acrylate, benzaldehyde, 4-methyl-Benzenemethanol, α,α-dimethyl-, phenanthrene, 2,6-Diisopropylnaphthalene
Ashrafi	2020	Wound Infection	10	Hexanal, 2-(2-Dodecoxyethoxy) ethanol, Octane, 1,1′-oxybis-, 1,2,3,6-Tetrahydropyridine, Glycerol/ Propane-1,2,3-triol, Linolenic acid, Adenosine, L- glutamine, 1,3-Dihydroxyacetone dimer
Monedeiro	2020	Visceral Adenocarcinoma	52 (total), 18/52 (cancer compounds)	**Healthy**: Octanal, 1-Octanol/Octanol/Isooctanol, 1-Hexanol, 2-ethyl-, 2-methyl dodecanoic acid, Heptanal, n-Pentanal, 2-Butenal, (E)-, Methacrolein, 4-methyl-2-heptanone, 2-phenoxyethanol, 7-Octen-2-ol, 2,6-dimethyl-/Dihydromyrcenol, Limonene, 1-undecene, Carbon disulfide, Tridecane<br>Furan, 2-pentyl-, Phenol, Benzyl alcohol, Acetic acid/Ethanoic acid, Toluene, Hexanal, Nonanal, Decanal, 2,4-dodecadienal, 2-Butanone, 5-Hepten-2-one, 6-methyl-, 6-Methyl-3,5-heptadiene-2-one, Geranylacetone/6,10-Dimethylundeca-5,9-dien-2-one, 2,4-dimethyl-3-pentanone, 2-butyl-cyclohexanone, 2,3-butanediol, 3,7-dimethyl-3-octanol, 3-methyl-1-butanol, Styrene, 4-methyl-1-hexene, 3,3-dimethyl-octene, 2-methylpentadecene, 5-methylhexen-2-ene, Acetonitrile, 2-methyl-pentane, 2,6,7-trimethyl-decane, 3-methyl-pentane, 6-methyl-tridecane, 5,8-diethyl-dodecane<br>n-Heptane, methyl cyclopentane, 2,4-dimethyl-pentane, 3-ethyl-pentane, Butanoic acid, Heptyl ester, Oxalic acid,

				allyl nonyl ester, 1,4-dimethylbenzene/xylene, Methyl benzoate, Ethylbenzene<br><br>**Cancer**: Octanal, 1-Octanol/Octanol/Isooctanol, 1-Hexanol, 2-ethyl-, 2-methyl dodecanoic acid<br>Heptanal, n-Pentanal, 2-Butenal, (E)-, Methacrolein, 4-methyl-2-heptanone, 2-phenoxyethanol<br>7-Octen-2-ol, 2,6-dimethyl-/Dihydromyrcenol, Limonene, 1-undecene, Carbon disulfide, Tridecane, Furan, 2-pentyl-, Phenol, Benzyl alcohol
Roodt	2018	Healthy	86	Octanal, 1-Octanol/Octanol/Isooctanol, 1-Hexanol, 2-ethyl-, Heptanal, 2-Butenal, (E)-, 7-Octen-2-ol, 2,6-dimethyl-/Dihydromyrcenol, Limonene, Phenol, Benzyl alcohol, Acetic acid/Ethanoic acid, Hexanal, Nonanal, Decanal, 5-Hepten-2-one, 6-methyl-, 6-Methyl-3,5-heptadiene-2-one, Geranylacetone/6,10-Dimethylundeca-5,9-dien-2-one, Dodecanoic acid, Tetradecanoic acid/Myristic Acid, Pentadecanoic acid, Heptadecanoic acid, 9,12-Octadecadienoic acid (Z,Z)-, 2-Undecanone, 1-Nonanol, Butyrolactone, Benzenemethanol, α,α-dimethyl-, Octanoic acid, Propanoic acid, Butanoic acid, Hexanoic acid, Heptanoic acid, Nonanoic acid, Decanoic acid, Tridecanoic acid, Hexadecanoic acid/n-hexadecanoic acid, Octadecanoic acid, Butanoic acid, 3-methyl-/Isovaleric acid, Hexanoic acid, 2-ethyl-, 2-Octenal, (E)-, 2-Nonenal, (E)-, 2-Decenal, (E)-<br>2-Undecenal, Furfural/2-Furancarboxyaldehyde, 2-Furancarboxaldehyde, 5-methyl-, 2-Heptanone<br>2-Octanone, 2-Nonanone, 2-Decanone, 2-Dodecanone, 2-Tridecanone, 2-Pentadecanone, 2-Hexadecanone, Acetophenone/1-phenyl-ethanone, 1-Hydroxy-2-butanone, Furyl hydroxymethyl ketone, 4H-Pyran-4-one, 2,3-dihydro-3,5-dihydroxy-6-methyl-, Ethanone, 1-(2-furanyl)-, Ethanone, 1-(4-methylphenyl)-, 2-Undecanone, 6,10-dimethyl-, 2,3-Pentanedione, Cyclopentanone, 2-Cyclopenten-1-one, Cyclopent-4-ene-1,3-dione, 2-Cyclopenten-1-one, 2-methyl-, 2-Cyclopenten-1-one, 3-methyl-, 1-Butanol, 2(3H)-Furanone, 5-methyl-, 2(5H)-Furanone<br>2(3H)-Furanone, 5-butyldihydro-, 2(3H)-Furanone, 5-hexyldihydro-, 2(3H)-Furanone, 5-heptyldihydro-, Linalool/3, 7- dimethyl-1,6-octadien-3-ol, α-Pinene, Benzonitrile, Pyrrole, Pyridine<br>Indole, 3-methyl-, 5,10-Diethoxy-2,3,7,8-tetrahydro-1H,6H-dipyrrolo[1,2-α:1′,2′-δ]pyrazine, Dimethyl sulfone, Dimethyl trisulphide, 1H-indole, Benzoic acid/Benzaldehyde, Benzeneacetaldehyde, Phenol, 2-methyl-, p-Cresol/4-methylphenol, Benzophenone, Benzothiazole
Mochalski	2018	Healthy	17	Heptanal, Acetic acid/Ethanoic acid, Hexanal, Benzoic acid/Benzaldehyde, 2-Propenal/Acrolein<br>Butanal, 3-methyl-, Propanal, 2-methyl-, 2-Ethacrolein, 2-Propanone/Acetone, 2-Pentanone<br>4-Methyl-2-pentanone, 2-Methyl-1-propanol, n-Butyl acetate, Ethyl formate, Ethyl propionate

				Vinyl butyrate, Ethyl isovalerate
Dolezal	2017	Healthy (all female)	137	Heptanal, Octanal, Limonene, Nonanal, Decanal, Tetradecanoic acid/Myristic Acid, Pentadecanoic acid, Heptadecanoic acid, Benzenemethanol, α,α-dimethyl-, Hexadecanoic acid/n-hexadecanoic acid, Octadecanoic acid, 2-Decanone, Acetophenone/1-phenyl-ethanone, α-Pinene, Tridecane, styrene, ethylbenzene, 2,6,10,15,19,23-hexamethyl-2,6,10,14,18,22-tetracosahexaene (squalene), 9-Octadecenoic acid, 13-Docosenoic acid, Dodecanal,, Tetradecanal, Undecanal, Octadecanal, Hexadecanal, Heptadecanal, Tridecanal, 4,8,12-Tetradecatrienal, 5,9,13-trimethyl-, 2,6-Di-tert-butyl-o-benzoquinone, 1-Hexadecanol, Octadecan-2-ol, 1-tetradecanol, Pentadecan-1-ol, n-Octacosan-1-ol, 1-Dodecanol, 1-Dodecanol, 2-methyl-<br>2-Methyl-1-undecanol, 2-Butoxyethanol, 1-Decanol, 2-ethyl- , 2-(2-Dodecoxyethoxy)ethanol<br>β-Pinene/6,6-dimethyl-2-methylene-, (IS)-Bicyclo[3.1.1]heptane, 1- dodecane, a- Springene, Docosenamide, Dodecane, Decane, Ethyl cyclohexane, 4-methyl octane, 3- methyl octane , Caffeine, Tocopherol, Octadecanoic acid, phenyl ester,<br>Tetradecanoic acid, undecyl ester<br>hexanedioic acid, octyl ester, Butan-2-yl 4-ethylbenzoate, Butyl pentanoate, 2,3-Diacetyloxypropyl<br>acetate (Triacetin), Butyl octanoate, Isopropyl dodecanoate, 2-Ethylhexyl benzoate/Benzoic acid, 2-ethylhexyl ester, Propan-2-yl tetradecanoate, 1,4-dioxacycloheptadecane-5,17-dione, 1,2,3-Propanetriol 2-octadecanoyl ester, 2,3-Dihydroxypropyl hexadecanoate, 2,3-Dihydroxypropyl 10-hydroxyoctadecanoate, 2,3-dihydroxypropyl 12-hydroxyoctadecanoate, Tetradecyl dodecanoate<br>Hexadecyl octanoate, 2,3-dihydroxypropyl octadec-9-enoate, Octadecyl 2-ethylhexanoate, Dodecyl tetradec-9-enoate, Tetradecyl tetradecanoate, Decyl octadecenoate, Dodecyl hexadec-9-enoate, Octadecyl tetradec-9-enoate, Hexyl octadec-9-enoate, Hexadecyl tetradecanoate<br>Tetradecyl hexadec-9-enoate, Octadecyl hexadec-9-enoate, Lauryl palmitoleate, Heptadecyl heptadecanoate, Icosyl octadecenoate, Dodecyl x-ester, 2-ethylhexyl 2-ethylhexanoate, Butyl hexanoate, Hexadecanoic acid, 1-methylethyl ester, Ethyl hexadecanoate/Palmitic acid, ethyl ester, Propan-2-yl hexadecanoate, Methyl octadecenoate, Butyl nonanoate, 1,2,3- triethyl 2-hydroxypropane- 1,2,3 tricarboxylate, cholesta-3,5-diene, Cholest-5-en-3-ol (3β)-, propanoate<br>Cholest-5-en-3-ol (3β)-, nonanoate, Cholest-5-en-3-ol (3β)-, tetradecanoate, Cholesta-4,6-dien-3-ol, (3β)-, Cholesterol, Cholest-7-en-3-ol, (3β,5α)- (Lathosterol), Lanost-8-en-3-ol, Benzene, 1,1′-(2-butene-1,4-diyl)bis-, Benzene, 1,1′-(3-methyl-1-propene-1,3-diyl)bis-, Benzene, 1-ethyl-2-methyl-, Benzene, (1-methylethyl)-, α-hexyl cinnamaldehyde, Phenol, 3,5-bis(1,1-dimethylethyl)-Naphthalene, Naphthalene, 1-methyl-, Epimanool

Duffy	2017	Healthy	35	Heptanal, Octanal, Nonanal, Decanal, Tetradecanoic acid/Myristic Acid, Pentadecanoic acid Hexadecanoic acid/n-hexadecanoic acid, 2,6,10,15,19,23-hexamethyl-2,6,10,14,18,22-tetracosahexaene (squalene), Dodecanal, Tetradecanal, Undecanal, Tridecanal, 1-Hexadecanol Acetic acid/Ethanoic acid, Hexanal, 1-Octanol/Octanol/Isooctanol, 1-Hexanol, 2-ethyl-, 5-Hepten-2-one, 6-methyl-, Dodecanoic acid, Nonanoic acid, Decanoic acid, 2-Decenal, (E)-, 2-Undecanone, 6,10-dimethyl-, Octane, 1,1′-oxybis-, Isopropyl Palmitate, Undecanoic acid, Glycine Lilial/α-methyl-β-(p-tert-butylphenyl)propanal, Tridecanol/1-tridecanol, Pentadecene, 1- Tridecene Tetradecane, Pentadecane, Propanoic acid, 2-methyl-, 1-(1,1-dimethylethyl)-2-methyl-1,3-propanediyl ester, N,N-dimethyl-1-hexadecanamine
Grabowska-Polanoska	2017	Healthy (mechanical disruption)	49	Heptanal, Octanal, Nonanal, Decanal, Acetic acid/Ethanoic acid, Hexanal, Limonene, Acetophenone/1-phenyl-ethanone, α-Pinene, Styrene, Ethylbenzene, Ethyl cyclohexane, Benzoic acid/Benzaldehyde, 2-Propenal/Acrolein, Propanal, 2-methyl-, 2- Propanone/Acetone, n-Butyl acetate, Phenol, Butyrolactone, 1-Butanol, n-Pentanal, Carbon disulfide, Toluene, 2-Butanone, Acetonitrile, n-Heptane, Methyl cyclopentane, 2,4-dimethyl-pentane, 1,4-dimethylbenzene/xylene n-Butanal, Acetaldehyde, 2-Propenal, 2-methyl-, Butanal, 2-methyl-, Ethanol/ethyl alcohol, 2-Propanol, 1,3-Pentadiene, 2-methyl-, (Z)-, 2-Butene, (E)-, Dimethyl sulfide, Nonane, Hexane, Cyclohexane, Methyl cyclohexane, 2,4-dimethylhexane, 2,3 dimethylheptane, Ethyl cyclopentane, Butane, Methyl acetate, Ethyl Acetate, Benzene
Martin	2016	Healthy (psychological stress)	4	Benzoic acid, n- decanoic acid, xylene (downregulated), 3- carene (downregulated)
Velhurst	2016	Healthy	23	Decanoic acid, Tetradecanoic acid/Myristic Acid, Pentadecanoic acid, Hexadecanoic acid/n-hexadecanoic acid, Dodecanoic acid, Nonanoic acid, 1-Dodecanol, 2-methyl, Geranylacetone/6,10-Dimethylundeca-5,9-dien-2-one, Octanoic acid, Hexanoic acid, Heptanoic acid, Tridecanoic acid, Butanoic acid, 3-methyl-/Isovaleric acid, Hexanoic acid, 2-ethyl-, 3-methyl-1-butanol, 9-hexadecanoic acid, Cedrol, Lactic acid, Isopropyl Myristate, Ethyl tetradecanoate, Ethyl citrate, Tributyl acetylcitrate, 1,1-dimethyl-3-phenyl propanol
Mochalski	2014	Healthy	64	Benzoic acid/Benzaldehyde, Heptanal, Octanal, Nonanal, Hexanal, 5-Hepten-2-one, 6-methyl-, Limonene, 2-Propenal/Acrolein, Propanal, 2-methyl-, 2-Propanone/Acetone, n-Butyl acetate, Butyrolactone, n-Pentanal, 2-Butanone, Acetonitrile, n-Heptane, n-Butanal, Acetaldehyde, 2-Propenal, 2-methyl-, Butanal, 2-methyl-, Ethanol/ethyl alcohol, 2-Propanol, 1,3-

				Pentadiene, 2-methyl-, (Z)-, 2-Butene, (E)-, Dimethyl sulfide, Nonane, Ethyl Acetate, β-Pinene/6,6-dimethyl-2-methylene-, (IS)-Bicyclo[3.1.1]heptane, Butanal, 3-methyl-, 2-Pentanone, 2-Butenal, (E)-, 2-Heptanone, Furan, 2-pentyl-, n- Propanal, 2-Hexenal, (E)-, 2-Butenal, 3-methyl-, Butanal, 2-ethyl-<br>2-Hexanone, 3-Buten-2-one/Methyl vinyl ketone, 3-Penten-2-one, 4-methyl-, p- cymene, Styrene, p,α-dimethyl-, Eucalyptol, 1-Octene, 1-Heptene, 2-Heptene, Isoprene, 1-Nonene, 2-Pentene, 2-methyl-, 1,3-Pentadiene, 2-methyl-, (E)-, Propene, 1-Pentene, 2-Butene, (Z)-, 2-Butene, 2,3-dimethyl-, Sulfide, allyl methyl, n-Octane, n-Pentane, Furan, 3-methyl-, Furan, 2-methyl-, 1,3-Dioxolane, 2-methyl-, Furan, 2,5-dimethyl-, 1,3-Dioxolane, Isopropyl acetate, Isobutyl acetate
Mochalski	2014	Healthy	12	Heptanal, Octanal, Nonanal, Hexanal, 5-Hepten-2-one, 6-methyl-, Limonene, 2- Propanone/Acetone, 2-Butanone, 2-Propenal, 2-methyl-, n- Propanal, 3-Buten-2-one/Methyl vinyl ketone, 2-Pentene, 2-methyl-
Broza	2014	Healthy	271	Heptanal, Octanal, Nonanal, Hexanal, 5-Hepten-2-one, 6-methyl-, Limonene, 2- Propanone/Acetone, 2-Butanone, Benzoic acid/Benzaldehyde, Propanal, 2-methyl-<br>n-Butyl acetate, n-Heptane, Butanal, 2-methyl- nonane, Ethyl Acetate, β-Pinene/6,6-dimethyl-2-methylene-, (IS)-Bicyclo heptane, Butanal, 3-methyl-, Eucalyptol, 1-Octene, Isoprene, 1-Nonene<br>n-Pentane, Geranylacetone/6,10-Dimethylundeca-5,9-dien-2-one, Isopropyl Myristate, Decanal<br>Acetic acid/Ethanoic acid, Dodecanal, Undecanal, Tridecanal, 1-Hexanol, 2-ethyl-, 2-Decenal, (E)-, Isopropyl Palmitate, Lilial/α-methyl-β-(p-tert-butylphenyl)propanal, Tetradecane, Pentadecane, Acetophenone/1-phenyl-ethanone, α-Pinene, Ethylbenzene, Carbon disulfide, Toluene, Methyl cyclopentane, 2,4-dimethyl-pentane, Hexane, Methyl cyclohexane, Benzene<br>1-Dodecanol, Dodecane, Decane, 4-Methyl-2-pentanone, 7-Octen-2-ol, 2,6-dimethyl-/Dihydromyrcenol, 1H-indole, 3,7-dimethyl-3-octanol, 3-methyl-pentane, Propanedioic acid<br>2-n-butylacrolein, 1,7,7-trimethylbicyclo(2;2;1)heptan-2-one, Tonalid, α-Isomethylionone, 2-methyl-5-(1-methylethenyl)-, (R)- 2-Cyclohexen-1-one, 1-(1-methylethoxy)-2-propanol<br>propylene glycol/1,2-propanediol, 2,2'oxybis-ethanol, 2-(2-hydroxypropoxy)-1-propanol<br>2,2-dimethyl-, diacetate 1,3-Propanediol, 2-(2-Hexyloxyethoxy)ethanol, (E)-3-Nonen-1-ol, 3,7-dimethyl-6-octen-1-ol, 3,7-dimethyl-1,6,octadien-3-ol, 3,7-dimethyl-,acetate 6-octen-1-ol, 3,7-dimethyl-,acetate 1,6-octadien-3-ol, 3,7-dimethyl-,acetate, (Z)-2,6-Octadien-1-ol, 5-methyl-2-(1-methylethenyl)-, acetate 4 hexen-1-ol, cyclohexanol,2-(1,1-dimethylethyl)-, 4-methyl-1-(1-methylethyl)- 3-Cyclohexen-1-ol, 1-methyl-4-(1-

				methylethenyl)-, acetate cyclohexanol, Oxacycloheptadec-8-en-2-one , p-menth-1-en-8-ol (alpha-terpineol), patchouli ethanol, veridiflorol, 1,3-pentadiene, 2,7-dimethyl-1,7-octadiene, 3,7-dimethyl-,(S)-1,6-Octadiene, 5-octadecene, 4-cyanocyclohexene, monoamide,N-(2-ethylhexyl)-pentyl ester oxalic acid, sulfur dioxide, sulfurous acid, hexyl octyl ester, 2-ethylhexyl hexyl ester Sulfurous acid, Octadecane Butane,2,2-dimethyl, Nonadecane, Dodecane,2,6,10-trimethyl-, 2-methyl butane, 3,4-dimethylheptane, 2-methylheptane, 3-methyl heptane, 2,2,4,6,6-pentamethyl-Heptane , 5-methyl- Tetradecane, 4-methyl-pentadecane, 2-bromotetradecane, 1-methanol, 3,3-dimethyl-2-(3-methyl-1,3-butadienyl)-Cyclohexane, 2-methyl-octadecane, 2-bromododecane, 2,2,4,4,6,8,8-heptamethylnonane, 1,2-dimethyl-cis-cyclopropane, 1,2-dimethyl-trans-cyclopropane, Butane, 1,1,2,3,4,4-hexachloro-tetrafluoro-, 2,3-dimethyl butane, (2-Methyl-propyl)-cyclohexane, 2,3-Dihydro-2,5-dimethyl-5H-1,4-dioxepin, l-2-chloro-Benzamide, N-tetrahydrofurfury 1-ethenyl-3-methylene-cyclopentene, 1-(hexyloxy)-5-methyl-hexane, Bicyclo[3.1.0]hexan-2-ol, 2-methyl-5-(1-methylethyl)-, (1.alpha.,2.alpha.,5.alpha.)- , 3,7-dimethyl-1,3,6-Octatriene, 1-methyl-4-(1-methylethyl)- 1,4-Cyclohexadiene, 1-methyl-2-octyl-cyclopropane, Aristolene, 1,2,3,4,5,6,7,8-octahydro-1,4,9,9-tetramethyl-, [1S-(1.alpha.,4.alpha.,7.alpha.)]- 4,7-Methanoazulene, 5,5 Dimethyl-3-vinyl cyclohex-2-en-1-one, 4,4-Dimethyl-2-pentyl methylphosphonofluoridate<br>Alpha, alpha.4-trimethyl- 3-Cyclohexene-1-methanol, 4-(2,6,6-trimethyl-2-cyclohexen-1-yl)-, (E)- 3-Buten-2-one, 4,7-Methano-1H-inden-6-ol, 3a,4,5,6,7,7a-hexahydro-, acetate, 1H-3a,7-Methanoazulene, octahydro-3,8,8-trimethyl-6-methylene-, [3R (3.alpha.,3a.beta.,7.beta.,8a.alpha.)]- , 1-(4-tert-Butylphenyl)propan-2-one, 3-(4-Isopropylphenyl)-2-methylpropionaldehyde, Cyclohexane, 1-ethenyl-1-methyl-2-(1-methylethenyl)-4-(1-methylethylidene)-, 4-(2,6,6-trimethyl-1-cyclohexen-1-yl)-, (E)- 3-Buten-2-one , 1,2-dimethyl-3,5-bis(1-methylethenyl)-Cyclohexane, Azulene, 1,2,3,4,5,6,7,8-octahydro-1,4-dimethyl-7-(1-methylethenyl)-, [1S-(1.alpha.,4.alpha.,7.alpha.)]- , 1,2,3,5,6,7-hexahydro-1,1,2,3,3-pentamethyl- 4H-Inden-4-one, 1-(2,6,6-trimethyl-2-cyclohexen-1-yl)- 1-Penten-3-one , 5-methyl-2-(1-methylethyl)-, cis- Cyclohexanone, Tricyclo[4.3.0.0(7,9)]nonane, 2,2,5,5,8,8-hexamethyl-, (1.alpha.,6.beta.,7.alpha.,9.alpha.)-, (1S)-Bicyclo[3.1.1]hept-2-ene, 6,6-dimethyl-2-[2-(phenylmethoxy)ethyl]-, 7-Acetyl-6-ethyl-1,1,4,4-tetramethyltetralin, tetradecyl oxirane<br>1-(1,2,3,4,7,7a-hexahydro-1,4,4,5-tetramethyl-1,3a-ethano-3aH-inden-6-yl)- Ethanone, 2,5-dihydro-furan, 2-Propenoic acid, 3-phenyl-, 2-methyl-2-propenyl ester, 12,15-octadecadiynoic acid, methyl ester, acetic acid, hexyl ester, isooctyl mercaptoacetate, 1,3-dimethylbutyl acetate

				bornyl acetate, 2-Deoxysorbitol pentaacetate, 2-ethylhexyl octyl ester Oxalic acid, Butyric acid, 1-methyl-1-p-tolylethyl ester, 2-methyl-, 1-(1,1-dimethylethyl)-2-methyl-1,3-propanediyl ester, Propanoic acid, 6-ethyloct-3-yl isohexyl ester Oxalic acid, 2-hydroxy-phenylmethyl ester benzoic acid, (2-phenyl-1,3-dioxolan-4-yl)methyl ester, trans- 9-Octadecenoic acid, 2-hydroxy-, pentyl ester Benzoic acid, 2-hydroxy-,3-methylbutyl benzoic acid, ethylene brassylate, 5-(1-Isopropenyl-4,5-dimethylbicyclo[4.3.0]nonan-5-yl)-3-methyl-2-pentenol acetate, 4-tert-butylcyclohexyl acetate, 3,N-dihydroxy-butanamide, 1-Propanamine, N,2-dimethyl-N-nitroso- , nopyl acetate, 4-(2,5, dimethyl-1H-pyrrol-1-yl)-N,N-dimethyl-Benzenamine, trichloromethane, 2-chloropropane, Benzene, 1,3-dimethyl, Benzene, 1,2,3-trimethyl-, Benzene, (1-butylheptyl)-, Benzene, (1-pentylheptyl)-, Benzene, 1,4-bis(1,1-dimethylethyl)- , (1-methylnonyl)-benzene, 1,1'-(3,3-dimethyl-1-butenylidene)bis- Benzene, (1-propyloctyl)- Benzene, (1-ethyloctyl)- Benzene, benzeneethanol,a,a-dimethyl-,1-acetate, Benzenemethanol, .alpha.-methyl-, acetate, 2H-1-Benzopyran, 3,5,6,8a-tetrahydro-2,5,5,8a-tetramethyl-, cis- , 1,3-didecyl- Benzene, o-xylene<br>1-methyl-2-(1-methylethyl)- Benzene, Butylated hydroxytoluene, alpha-cubebene, ambrox, 6,7-diethyl-1,2,3,4-tetrahydro-1,1,4,4-tetramethyl- Naphthalene, 1,3,4,6,7,8-hexahydro-4,6,6,7,8,8, Hexamethyl-cyclopenta-gamma-2-benzopyran/galaxolide, 1,3-diol monopropionate Indan
Kusano	2013	Healthy	35	Heptanal, Octanal, Nonanal, Benzoic acid/Benzaldehyde, Geranylacetone/6,10-Dimethylundeca-5,9-dien-2-one, Decanal, Dodecanal, Undecanal, 1-Hexanol, 2-ethyl-, Tetradecane, Pentadecane, Acetophenone/1-phenyl-ethanone, Dodecane, Octadecane, Dodecanoic acid, Hexanoic acid, Tetradecanal, Pentadecene, Phenol, 2-Decanone, Tridecane, Benzyl alcohol, 2-Nonenal, (E)-, Furfural/2-Furancarboxyaldehyde, Linalool/3, 7-dimethyl-1,6-octadien-3-ol, Phenylpropanoic acid, Heptadecene, Undecane, Hexadecane, Eicosane, Tricosane, Docosane, Decanoic acid, methyl ester, Methyl 9-methyltetradecanoate, Octanoic acid, methyl ester
Abaffy	2013	Melanoma	6	Dodecanoic acid, Toluene, Tetradecanoic acid/Myristic Acid, Hexadecanoic acid/n-hexadecanoic acid, 1-eicosane, 2- ethylhexyl- 4- methoxy- cinnamate
Dormont	2013	Healthy (compared 4 techniques)	44	Nonanal, Decanal, 5-Hepten-2-one, 6-methyl-, Benzoic acid/Benzaldehyde, Octanal, Geranylacetone/6,10-Dimethylundeca-5,9-dien-2-one, Nonane, Tetradecane, Heptadecene<br>Pentadecane, Hexadecane, Dodecanal, 2,6,10,15,19,23-hexamethyl-2,6,10,14,18,22-tetracosahexaene (squalene), Decane, Nonanoic acid, Tetradecene, Hexanal, Dodecane, Undecane, 2-Nonenal, (E)-, Tridecane, α-Pinene, Undecanal, Limonene, Pentadecene, Linalool/3,

				7- dimethyl-1,6-octadien-3-ol, Tridecanal, Ethyl Acetate, Methyl octadecenoate, Diethylsulfide, Methyl hexadecanoate, Octadecyl acetate<br><br>Hexadecene, Decanoic acid, Octanoic acid, 1-Dodecanol, Lilial/α-methyl-β-(p-tert-butylphenyl)propanal , 2-Decenal, (E)-, 2-Octenal, (E)-, Benzothiazole, (E)-2-Undecenal<br>(E)-2-Heptenal, (E,E)-2,4-Nonadienal, Nonalactone
Ruzsanyi	2012	Healthy	10	Nonanal, Decanal, 5-Hepten-2-one, 6-methyl-, Benzoic acid/Benzaldehyde, Octanal, Ethanol/ethyl alcohol, 1-Hexanol, 2-ethyl-, Ammonia, n-Butyl acetate, 2-Butenal, 3-methyl-
Prada	2011	Healthy	51	Octanal, Nonanal, Benzoic acid/Benzaldehyde, Geranylacetone/6,10-Dimethylundeca-5,9-dien-2-one, Decanal, Dodecanal, Undecanal, Tetradecane, Pentadecane, Dodecane, Pentadecene, Tridecane, 2-Nonenal, (E)-, Linalool/3, 7- dimethyl-1,6-octadien-3-ol, Heptadecene, Undecane, Hexadecane, Hexanal, 5-Hepten-2-one, 6-methyl-, Limonene, Nonane, Ethyl Acetate, Tridecanal, α-Pinene, Decane, Nonanoic acid, 2,6,10,15,19,23-hexamethyl-2,6,10,14,18,22-tetracosahexaene (squalene), Methyl octadecenoate, Tetradecene, Diethylsulfide, Methyl hexadecanoate, Octadecyl acetate, 2-Decenal, (E)-, Lilial/α-methyl-β-(p-tert-butylphenyl)propanal , 1-Dodecanol, Decanoic acid, Octanoic acid, 2-Octenal, (E)-, Benzothiazole, (E)-2-Undecenal, (E)-2-Heptenal, (E,E)-2,4-Nonadienal, Nonalactone, Hexadecene
Abaffy	2010	Melanoma	9	Octanal, Nonanal, Decanal, Hexanal, Limonene, Heptanal, Ethylbenzene, 2-Ethylhexyl benzoate/Benzoic acid, 2-ethylhexyl ester, 1-Methyl-bis(1,2,4)-triazole-5,1'
Thomas	2010	Wound Infection	46	Nonanal, Geranylacetone/6,10-Dimethylundeca-5,9-dien-2-one, 1-Hexanol, 2-ethyl-, Tetradecanal, Propylene glycol/1,2-propanediol, Butylated hydroxytoluene, Carene/3-carene<br>Phenol, 3,5-bis(1,1-dimethylethyl)-, 1-eicosanol, 2-hexadecanol, 1-Dodecanol, 3,7,11-trimethyl- , 1,3-Pentanediol, 2,2,4-trimethyl- , 1-decanol,2-hexyl, 1-Propyl-3,6-diazahomoadamantan-9-ol, 2-Propanol, 1-(2-methoxy-1-methylethoxy)- , 3-decen-1-ol, E-2-Tetradecen-1-ol , Z,Z-2,5-Pentadecadien-1-ol, Cyclodecanol, 2-t-Butyl-5-propyl-[1,3]dioxolan-4-one, 1-nonadecene, 2-Methyl-Z-4-tetradecene, 7-tetradecene, Disulfide, dimethyl, Dodecane, 1,2-dibromo-, 3-Isopropyl-6a,10b-dimethyl-8-(2-oxo-2-phenyl-ethyl)-dodecahydro-benzo[f]chromen-7-one, 2-propanol, 1-(1-methyethoxy), 1,4-Methanoazulen-3-ol decahydro-1,5,5,8a-tetramethyl-, [1s-(1.a.,3.b.,3a.b.,4.a.,8a.b.)]-, a.-Ethylether of 11-epi-dihydroartemisinin, 4-Trifluoroacetoxypentadecane, 4-Trifluoroacetoxytetradecane, 2-Trifluoroacetoxypentadecane

				Propanoic acid, 2-methyl-, 3-hydroxy-2,4,4-trimethylpentyl ester, E-2-Methyl-3-tetradecen-1-ol acetate, Pentanoic acid, 2,2,4-trimethyl-3-carboxyisopropyl, isobutyl ester, 1,2-Benzenedicarboxylic acid, butyl 2-ethylhexyl ester, 2,6-Nonadienoic acid, 7-ethyl-9-(3-ethyl-3-methyloxiranyl)-3-methyl-, methyl ester, [2R-[2.a.(2E,6E),3.a.]-, Myristic acid, 9-hexadecenyl ester, (Z)-, Octadecanoic acid, 4-hydroxy-, methyl ester , Cyclopropaneoctanoic acid, 2-[(2-pentylcyclopropyl)methyl]-, methyl ester, trans,trans-, Z-3-Octadecen-1-ol acetate<br>2-Indazol-2-ylphenylamine, Octadecane,1-chloro , 1,3,5-Tris(trimethylsiloxy)benzene , 3-Benzoylmethyl-3-hydroxy-5-nitro-2-indolinone , 3-tert-butyl-5-chloro-2-hydroxybenzophenone
Turner	2008	Healthy	6	2- Propanone/Acetone, Acetaldehyde, Ethanol/ethyl alcohol, Methanol, Propanol, Ammonia
Gallagher	2008	Healthy	90	2- Propanone/Acetone, Nonanal, Geranylacetone/6,10-Dimethylundeca-5,9-dien-2-one, Octanal<br>Decanal, Benzoic acid/Benzaldehyde, Dodecanal, Linalool/3, 7- dimethyl-1,6-octadien-3-ol, 5-Hepten-2-one, 6-methyl-, Acetophenone/1-phenyl-ethanone, Hexanoic acid, Phenol, Acetic acid/Ethanoic acid, Lilial/α-methyl-β-(p-tert-butylphenyl)propanal , 7-Octen-2-ol, 2,6-dimethyl-/Dihydromyrcenol, 1,7,7-trimethylbicyclo(2;2;1)heptan-2-one, 3,7-dimethyl-6-octen-1-ol , p-menth-1-en-8-ol (alpha-terpineol), 4-tert-butylcyclohexyl acetate, 1,3,4,6,7,8-hexahydro-4,6,6,7,8,8-hexamethyl-cyclopenta-gamma-2-benzopyran/galaxolide, p- cymene, Octanoic acid, Butanoic acid, 3-methyl-/Isovaleric acid, Hexanoic acid, 2-ethyl-, cedrol, 2-ethylhexyl 2-ethylhexanoate, Hexadecanoic acid, 1-methylethyl ester, α-hexyl cinnamaldehyde, Propanoic acid, Butanoic acid<br>Pyridine, Dimethyl sulfone, p-Cresol/4-methylphenol, 4-cyanocyclohexane, 2-(4-tert-butylphenyl) propanal , 5-(hydroxymethyl)-2-furaldehyde, 3,5,5-trimethylcyclohex-2-en-1-one (isophoron), 1-(2-methoxypropoxy)-2-propanol, 1,1′-oxybis-2-propanol, 1-octen-3-ol, 3,7-dimethyl-2,6-octadien-l-ol<br>2-(2-propyl)-5-methyl-1-cyclohexanol<br>(menthol) /Cyclohexanol, 5-methyl-2-(1-methylethyl)-, [1R-(1.alpha.,2.beta.,5.alpha.)]-, Furfural alcohol/2-furanmethanol, 1-phenylethylester acetic acid, Tetradecanoic acid, 1-methylethyl ester, 1,3-diacetyloxypropan-2-yl acetate, 2-hydroxy, hexyl ester benzoic acid , Methyl 2-pentyl-3-oxo-1-cyclopentyl acetate, Methoxy acetic acid, dodecyl ester, Homomenthylsalicylate, 2-ethylhexylsalicylate , 2,4,6-tri-tert-butyl-phenol, Phenyl ethyl alcohol/phenylethanol, 2,6-bis(1,1-dimethylethyl)-4-(1-oxopropyl)phenol, 1-Hexanol, 2-ethyl-<br>2,6,10,15,19,23-hexamethyl-2,6,10,14,18,22-tetracosahexaene (squalene), Dodecanoic acid,

				Tetradecanoic acid/Myristic Acid, Hexadecanoic acid/n-hexadecanoic acid, Pentadecanoic acid<br>Tridecanoic acid, 9-hexadecanoic acid, Lactic acid, 1-Butanol, Heptadecanoic acid, Ethyl hexadecanoate/Palmitic acid, ethyl ester, 6-Methyl-3,5-heptadiene-2-one, 2-Cyclopenten-1-one, 2-methyl-, 2-Cyclopenten-1-one, 3-methyl-, Benzothiazole, glycerol/Propane-1,2,3-triol , 2,5-hexanedione, 6-hydroxy-hexan-2-one, 3- hexanol, 2- hexanol, 1,6-heptadien-4-ol, 3-hexene-2,5-diol, 3-methyl-cyclopentanol, 1-methyl-cyclopentanol, 2-methyl-cyclopentanol, 2(5H)-furanone, 3-methyl, 1-methoxy-hexane, Ethyl carbamate , 4-ethyl-morpholine, Lactic acid, methyl ester<br>1-methyl hexyl acetate, Methoxy acetic acid, tetradecyl ester, Hexadecanoic acid, 2-hydroxyethylester, Ethyl (-)-lactate, 4-vinyl imidazole
Curran	2007	Healthy	62	Nonanal, Geranylacetone/6,10-Dimethylundeca-5,9-dien-2-one, Decanal, Pyridine, Tetradecanoic acid, 1-methylethyl ester, Octanal, Benzoic acid/Benzaldehyde, Dodecanal, Linalool/3, 7- dimethyl-1,6-octadien-3-ol, 5-Hepten-2-one, 6-methyl-, Phenol, 3,7-dimethyl-6-octen-1-ol , Furfural alcohol/2-furanmethanol, Phenyl ethyl alcohol/phenylethanol, Tetradecanal, Hexanal, Heptanal, Undecanal, Tetradecane, Pentadecane, Dodecane, Tridecane, 2-Nonenal, (E)-, Heptadecene, Undecane, Hexadecane, Nonane, Tridecanal, Toluene, 2-Decanone, Benzyl alcohol, Furfural/2-Furancarboxyaldehyde, Eicosane, Decanoic acid, Methyl ester, Octanoic acid, Butanal, 2-methyl-, β-Pinene/6,6-dimethyl-2-methylene-, (IS)-Bicyclo[3.1.1]heptane, 2-Decenal, (E)-Benzene, 1,3-dimethyl, Benzene, 1,2,3-trimethyl-, n-Octane, Decanoic acid, 1-Octanol/Octanol/Isooctanol, 1,4-dimethylbenzene/xylene, Benzene, 1-ethyl-2-methyl-, Naphthalene, 1-Nonanol, 2-Octenal, (E)-, Cyclohexadecane, Cyclotetradecane, 7-Hexadecenoic acid, methyl ester, Dodecanoic acid, methyl ester, Furancarboxylic acid-methyl ester, Hexadecanoic acid, methyl ester, Hexanedioic acid, dimethyl ester, Propanedioic acid, dimethyl ester, Nonanoicacid, methyl ester, 1-chlorononane, Thiazolidine, Benzene, 1,3,5-trimethyl, Benzene,1-ethyl-3-methyl-, 1,2,4-Trimethylbenzene
Zhang	2005	Healthy	34	Nonanal. Decanal, Octanal, Benzoic acid/Benzaldehyde, 3,7-dimethyl-6-octen-1-ol , Phenyl ethyl alcohol/phenylethanol, Tetradecane, Pentadecane, Dodecane, Tridecane, Undecane, Hexadecane, Eicosane, Acetophenone/1-phenyl-ethanone, Lilial/α-methyl-β-(p-tert-butylphenyl)propanal, Cedrol, 3,7-dimethyl-2,6-octadien-l-ol , 1-Hexanol, 2-ethyl-, Limonene, Pentadecene, α-Pinene, Methyl salicylate, 5-Methyl-2-isopropyl cyclohexanol, 1,3,5,7-Cyclooctene, Camphene, 3-Dodecene, Caryophyllene, Hexadecene, 2-Methyl dodecane

				Cyclopentadecane, 2-Methyl hexadecane, Isobornyl propionate, Tetramethyl thiourea, Diphenyl ether
Curran	2005	Healthy	46	Nonanal, Decanal, Octanal, Benzoic acid/Benzaldehyde, Tetradecane, Dodecane, Tridecane, Undecane, α-Pinene, Hexadecene, Pyridine, Tetradecanoic acid, 1-methylethyl ester, 5-Hepten-2-one, 6-methyl-, Phenol, Furfural alcohol/2-furanmethanol, Tetradecanal, Hexanal, Heptanal, Undecanal, 2-Nonenal, (E)-, Heptadecene, Nonane, Toluene, Benzyl alcohol, Furfural/2-Furancarboxyaldehyde, Decanoic acid, methyl ester, Octanoic acid, methyl ester, Naphthalene 7-Hexadecenoic acid, methyl ester, Dodecanoic acid, methyl ester, Furancarboxylic acid-methyl ester, Hexadecanoic acid, methyl ester, Hexanedioic acid, dimethyl ester, Propanedioic acid, dimethyl ester, 1-chlorononane, 1-phenylethylester acetic acid, Dodecanoic acid, Tetradecanoic acid/Myristic Acid, Methyl 9-methyltetradecanoate, 2-Undecanone, 6,10-dimethyl-, Methylpentadecanoic acid, Nonanoic acid, methyl ester, Tridecanoic acid, methyl ester Cyclopentanetridecanoic acid–methyl ester, Hexanoic acid–methyl ester, Undecanoic acid–methyl ester
Haze	2001	Healthy	22	Nonanal, Decanal, Octanal, Dodecane, Undecane, Hexanal, Heptanal, 2-Nonenal, (E)-, Nonane 1-Hexanol, 2-ethyl-, 1-Octanol/Octanol/Isooctanol, Acetic acid/Ethanoic acid, 1-Butanol, 4-Methyl-2-pentanone, 1-Hexadecanol, Octadecanol, Butyric acid , 6- methyl-5-heptanone, 1-hexanol, 1-Decanol, Amyl alcohol/1-pentanol, 4-methyl-2-pentene
Meijerink	2000	Healthy	75	Nonanal, Decanal, Octanal, Hexanal, Heptanal, 1-Octanol/ Octanol/ Isooctanol, Acetic acid/Ethanoic acid, 1-Butanol, 1-Hexadecanol, 1-hexanol, 1-Decanol, amyl alcohol/1-pentanol, Pyridine, 5-Hepten-2-one, 6-methyl-, Furfural alcohol/2-furanmethanol, Benzyl alcohol, Furfural/2-Furancarboxyaldehyde, Phenyl ethyl alcohol/phenylethanol, Geranylacetone/6,10, Dimethylundeca-5,9-dien-2-one, 1-Nonanol, 2-Propanone/Acetone, Hexanoic acid, Butanoic acid, 3-methyl-/Isovaleric acid, Hexanoic acid, 2-ethyl-, Propanoic acid, Butanoic acid, 2-(2-propyl)-5-methyl-1-cyclohexanol (menthol) /Cyclohexanol, 5-methyl-2-(1-methylethyl)-, [1R-(1.alpha.,2.beta.,5.alpha.)]-, 6-Methyl-3,5-heptadiene-2-one, Ethanol/ethyl alcohol, 1-Dodecanol 1H-indole, 2-Propanol, 3-Penten-2-one, 4-methyl-, 3-methyl-1-butanol, ethyl tetradecanoate 1-tetradecanol, 2-Nonanone, Benzonitrile, 3-hydroxy-2-butanone, Cyclohexanone, 1-heptanol 2-methyl-1-butanol, 3,5,5-trimethyl-1-hexanol, 1-methoxy-2-propanol, 3-methyl-2-buten-1-ol 4-methyl-4-penten-2-ol, 3-ethyl-2,2-dimethyloxirane, linalooloxide, linalooloxide A, Nicotine, Ethyl butanoate, Ethyl 2-methylbutanoate, Ethyl 3-methylbutanoate,

				Ethyl isohexanoate, 1-methoxy-2-propyl acetate, Ethyl hexanoate, Ethyl 4-methylhexanoate, Ethyl heptanoate, Ethyl 4-methylheptanoate, Ethyl octanoate, Ethyl 4-methyloctanoate, Ethyl nonanoate, Ethyl decanoate, Ethyl 4-methyldecanoate, dec-9-enoic acid, ethyl estert, Ethyl undecanoate, Ethyl 4, Methylundecanoate, Ethyl isododecanoate, Ethyl dodecanoate, Ethyl 4-methyldodecanoateo, Ethyl isotridecanoate, Ethyl isotetradecanoate, Ethyl 9-tetradecenoate, Ethyl pentadecanoate, Ethyl 9-hexadecenoate
Bernier	2000	Healthy	215	Nonanal, Decanal, Acetic acid/Ethanoic acid, 1-Decanol, Pyridine, 5-Hepten-2-one, 6-methyl-, Furfural alcohol/2-furanmethanol, Benzyl alcohol, Phenyl ethyl alcohol/phenylethanol, Geranylacetone/6,10-Dimethylundeca-5,9-dien-2-one, Hexanoic acid, Propanoic acid, 6-Methyl-3,5-heptadiene-2-one, 1H-indole, 1-tetradecanol, 4-methyl-4-penten-2-ol, Nonane, 4-methyl-2-pentene, Benzoic acid/Benzaldehyde, Tetradecane, Hexadecene, Tetradecanoic acid, 1-methylethyl ester, Phenol, Heptadecene, Toluene, Octanoic acid, methyl ester, Hexadecanoic acid, methyl ester, Hexanedioic acid, dimethyl ester, 1-chlorononane, Dodecanoic acid, Tetradecanoic acid/Myristic Acid, Methylpentadecanoic acid, Nonanoic acid, methyl ester tridecanoic acid, methyl ester, 3,7-dimethyl-6-octen-1-ol , Pentadecane, Hexadecane, 3,7-dimethyl-2,6-octadien-l-ol , Dodecanal, 2-Decanone, n-Octane, Decanoic acid, 1,4-dimethylbenzene/xylene, Cyclohexadecane, Cyclotetradecane, Thiazolidine, 1-octen-3-ol<br>Octanoic acid, 2,6,10,15,19,23-hexamethyl-2,6,10,14,18,22-tetracosahexaene (squalene), Hexadecanoic acid/n-hexadecanoic acid, Pentadecanoic acid, Tridecanoic acid, Lactic acid, Heptadecanoic acid, Glycerol/Propane-1,2,3-triol , 2-hexadecanol, Ethylbenzene, Decane<br>Nonanoic acid, Tetradecene, Octadecane, Tricosane, Docosane, n-Heptane, Carbon disulfide , Hexane, Benzene, Dimethyl sulfide, 2-Pentanone, 2-Hexanone, 1-Pentene, Furan, 3-methyl-<br>Furan, 2-methyl-, Heptanoic acid, Undecanoic acid, Tridecanol/1-tridecanol, N,N-dimethyl-1-hexadecanamine, Styrene, 2,4-dimethylhexane, Octadecanoic acid, Octadecanoic acid, phenyl ester, Tetradecanoic acid, undecyl ester,<br>Hexanedioic acid, octyl ester<br>cholesta-3,5-diene, 2-methyl dodecanoic acid, Docosanoic acid, Heptanedioic acid, 2- propenoic acid , 2-butenoic acid, 2-methyl-2-butenoic acid, 3-methyl-2-pentenoic acid, 3-methylpentanoic acid, Methyltridecanoic acid, Methylheptadecanoic acid, Hexanedioic acid, 4-hydroxy-3-methoxybenzoic acid, 9-Hexadecenoic acid, 2-methylhexadecanal, Butanone, 3-, Pentanone , 2-nonen-4-one, 2- butanol, 2-decanol, 2-heptadecanol, 2-methyl-3-pentanol, Ethylene glycol, 2-hexen-1-ol, 4-hexen-1-ol, 1-hexen-3-ol, 1-hepten-3-ol, 2-octen-1-ol, 2-methyl-3, Octenol, Nonenol, Dodecenol,

				Methyldodecene, 2-methyl-2-dodecene, 3-ethyl-1,4-hexadiene<br>2,6-dimethyl-1-heptene, 2-methyl-1-heptene, 4-ethyl-3-heptene, 2-methyl-1-hexene, 2-methyl-2-undecene, 3-methyl-5-undecene, 4-methyl-4-undecene, 9- octadecene, 2-octene, 3- octene, 4- octene, 3,4-nonadiene, Trimethyl-1,5-heptadiene, 2,7-dimethyl-1-octene, 5-decene, Undecadiene<br>4- nonene, Dimethylpentadiene, Methylheptadecane, Trimethyl-3-methylenehexadecane, Heneicosane, Pentacosane, Menthane, N-menth-6-ene, 2-(2H-benzotriazol-2-yl)-4-methylphenol, 2-<br>butenoic acid, butyl ester, Butanoic acid, methyl ester, 13-methylpentadecanoic acid, methyl ester, 14-methylpentadecanoic acid, methyl ester,<br>Octadecenoic acid, methyl ester,<br>Hexadecanoic acid, butyl ester,<br>Tetracosanoic acid, methyl ester<br>pentanedioic acid, ester, Pentanedioic acid, mono(2-ethylhexyl) ester, Heptanedioic acid, dibutyl ester, Hydroxybutanoic acid, ethyl ester, 3-hydroxybenzoic acid, methyl ester, 4-hydroxybenzoic acid, propyl ester, 2-hydroxybenzoic acid, phenylmethyl ester, 2,4-dihydroxy-3,6-dimethylbenzoic acid, 16-methylheptadecanoic acid, methyl ester, Propanamide, N,N-diethyl-3-methylbenzamide (DEET)<br>N,N-bis(2-hydroxyethyl)dodecanamide, N,N-didodecyl formamide, 1,3-butanediamine, N,N-dimethyl-1,2-ethanediamine, N-ethylcyclopentamine, N,N-dimethyl-3-butoxypropylamine, N,N-dimethyl-3-benzyloxypropylamine, N-methyl-N-nitroso-1-dodecanamine, N,N-dimethyl-1-dodecanamine, N,N-dimethyl-1-tridecanamine, N,N-dimethyl-1-tetradecanamine, N,N-dimethyl-1-pentadecanamine, N,N-dimethyl-1-heptadecanamine, N,N-dimethyl-1-octadecanamine<br>methyl iodide, 1-chlorohexane, 1-chloroheptane, 1-chlorododecane, 1-chlorotetradecane, 1-chloropentadecane, 1-chlorohexadecane, 2-methyl-1H-pyrrole, 3-methyl-1H-pyrrole, 3-pyridinamine, 4-pyridinamine, 4-methyl-2-pyridinamine, 2-methylpyridine, 4(1H)-pyridinone, 6-amino-3-pyridine carboxylic acid, 3(1-methyl-2-pyrrolidinyl)pyridine (nicotine), 1,2,3-4-tetrahydroquinoline, 2,3,4-trimethylquinoline, 2-ethylpiperidine, 1-piperidineethanol, 4-piperidinemethanamine, 1-phenyl-3-(1-piperidinyl)-2-buten-1-one, 4,5-dihydro-2-methyl-1H-imidazole, 1,5-dimethyl-1H-pyrazole, pyrazine, 2,6-dimethylpyrazine, trimethylpyrazine, 2-methylisothiazole, Thiomethane, 2-thiopropane, 1-methylthiobutane, 1-thiododecane, 3-methylthietane, 3-(methylthio)-1,2-propanediol, o-(2-butenylthio)phenol, cholest-5-en-3-ol, 4-hydroxybenzoic acid, 3-hydroxy-4-methylbenzaldehyde, 4-phenylmethoxybenzaldehyde, propylbenzene, 4,4′-dimethyl-1,1′-biphenyl
